# Supplementary material for: Attentional modulation of secondary somatosensory and visual thalamus of mice
Source: bioRxiv. 2024 Mar 25:2024.03.22.586242. Preprint. [Version 1] doi: 10.1101/2024.03.22.586242 (PMC10996504; doi:10.1101/2024.03.22.586242)

Figure S1: Manual curation of POm and LP cells.

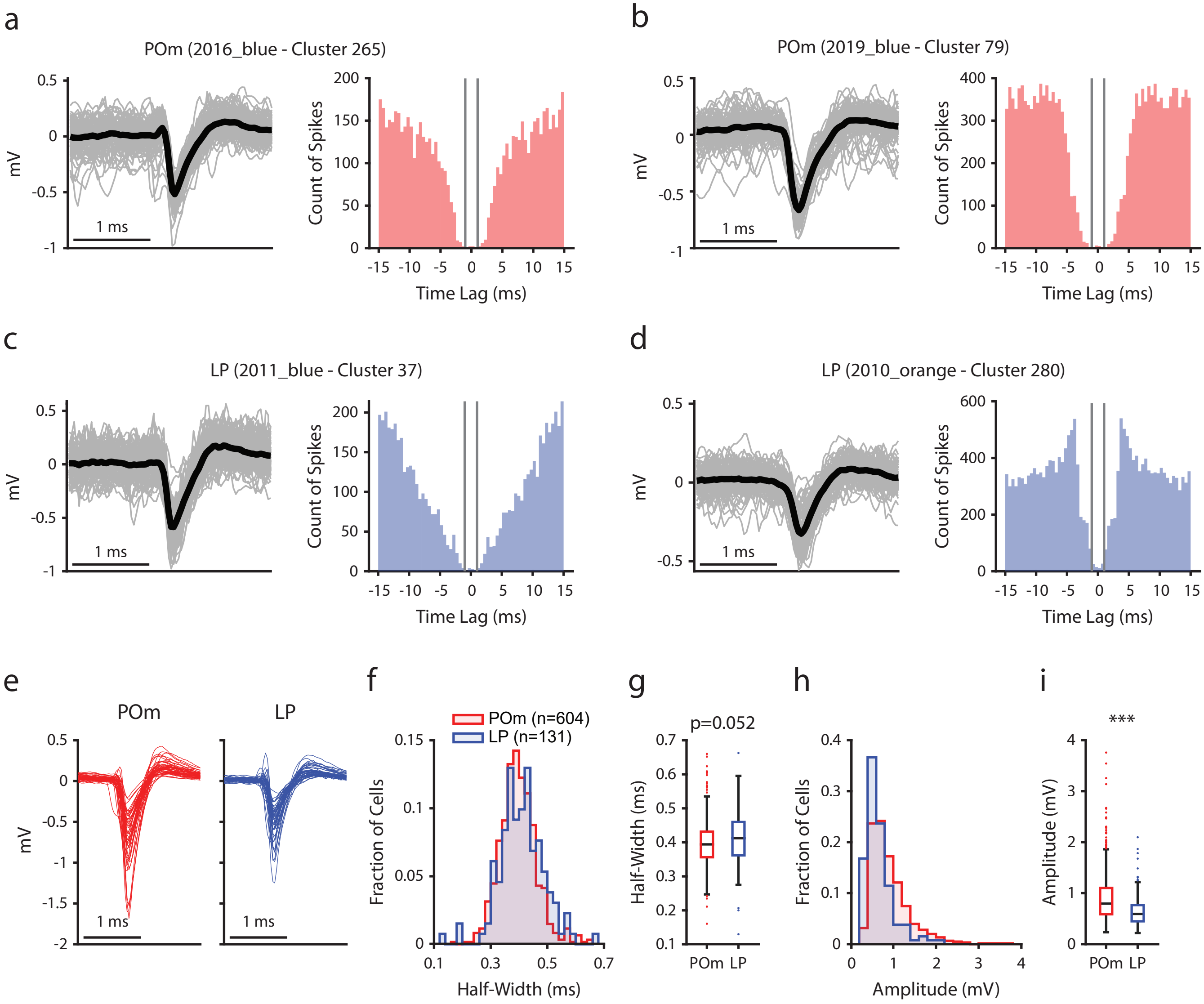

Figure S2: Conditioning alters sensory response latency in POm and LP

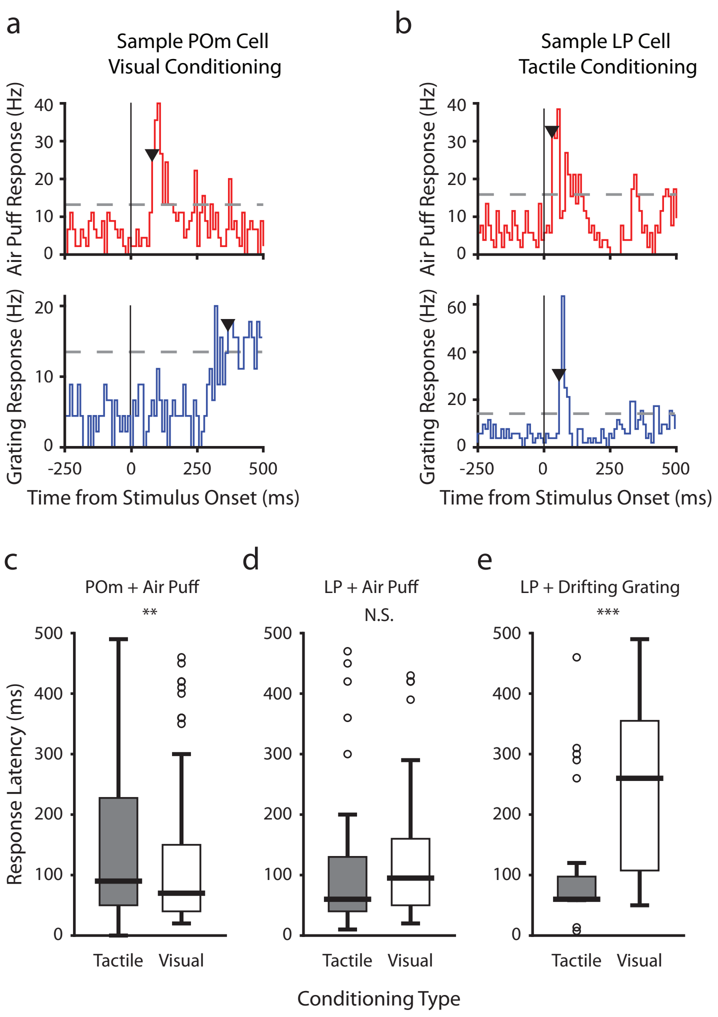

Figure S3: LP responses to tactile and visual stimuli as a function of anatomical location

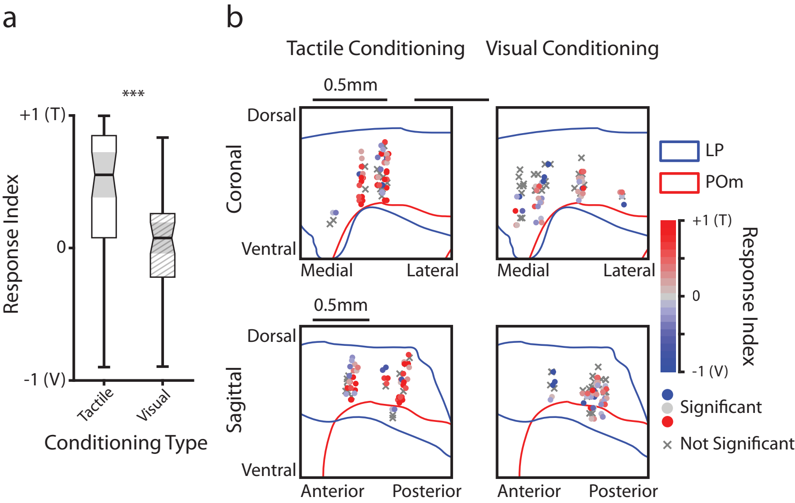

Supplement: 1 — Figure S1: Manual curation of POm and LP cells a, b, Example POm cells. Left, Mean spike waveform (black) with 200 randomly sampled individual waveforms (gray). Right, Autocorrelogram, binned at 0.5ms. Gray lines indicate a time lag of +/−1ms. c, d, Example LP cells, as in a and b. e, Mean waveforms of 50 randomly selected POm cells (red) and 50 randomly selected LP cells (blue). f, Histogram of waveform half-widths of each recorded POm cell (red, n=604) and LP cell (blue, n=131). g, Box chart of waveform half-width. Half-widths were not significantly different between LP and POm cells (POm: 0.40+/−0.07ms, LP: 0.41+/−0.08ms (mean+/−std), t-test p=0.052). h, Histogram of waveform amplitudes from each recorded POm and LP cell. i, Box chart of waveform amplitudes. POm cells had significantly higher amplitudes than LP cells (POm: 0.91+/−0.47mV, LP: 0.67+/−0.33mV (mean+/−std), t-test p<0.001). Figure S2: Conditioning alters sensory response latency in POm and LP a, b, Sample sensory response measurements of a POm cell from a visually conditioned mouse (a) and a LP cell from a tactilely conditioned mouse (b). A cell’s response latency was measured as the first time the mean firing rate exceeded a 99% confidence interval for at least two 10 ms time bins. Red: firing rate aligned to air puff onset. Blue: firing rate aligned to drifting grating onset. Dotted gray line: 99% confidence interval of baseline firing rate. Black triangle: response latency. Cells that did not respond to a stimulus within 500 ms were excluded from further analysis. c, Box chart of POm response latency to the air puff after tactile conditioning (gray) and visual conditioning (white). The air puff response latency was shorter in visually conditioned mice (n=219 responding cells from tactilely conditioned mice, 130 from visually conditioned mice; p<0.005, Wilcoxon rank-sum test). d, LP response latency to the air puff. There was no difference in response latency to the air puff between tactilely and vi [file NIHPP2024.03.22.586242V1-supplement-1.pdf]
